# Elementary Treatise on Practical Chemistry and Qualitative Inorganic Analysis

**Published:** 1881-04

**Authors:** 


### BIBLIOGRAPHICAL.

---

*Elementary Treatise on Practical Chemistry, and Qualitative Inorganic Analysis.* Adapted for Laboratories of Colleges, Schools and Beginners. By Frank Clowes, C. Sc., London, 370 pages. Philadelphia, Henry C. Lea's Sons & Co. 1881.

This is a working book, intended for the use of those who have been made familiar with the nomenclature and foundation work of chemical science, and presupposes a knowledge of the first principles. To those who propose to do laboratory chemistry—and there is really no other way of learning it,—such a book is valuable as containing sufficiently minute directions as to manipulation, &c. The key-note of the book is had in the words we copy from page 86 on the method of trying the chemical analytical reactions: "The student must consider it a necessary condition of after success that each result is obtained precisely as stated in the text, and must never on any account pass on until he has conscientiously satisfied himself that the statements of the book are true, and that he could at any time repeat the text successfully." The number of text books of chemistry issuing from time to time indicates a demand for such literature, and that the number of students is augmenting. With this book much may be learned of manipulation, (which is *real* knowledge) as against simple theory (which is *ideal* knowledge only.) If our dental students would take such books as this and perform a few experiments for themselves, they would acquire a practical knowledge of chemistry which would make the chemical lectures less tedious and more lucid.

